# The USP21 Short Variant (USP21SV) Lacking NES, Located Mostly in the Nucleus *In Vivo*, Activates Transcription by Deubiquitylating ubH2A *In Vitro*

**DOI:** 10.1371/journal.pone.0079813

**Published:** 2013-11-22

**Authors:** Hiroshi Okuda, Hideki Ohdan, Manabu Nakayama, Haruhiko Koseki, Takeya Nakagawa, Takashi Ito

**Affiliations:** 1 Department of Biochemistry, Nagasaki University School of Medicine, Nagasaki, Japan; 2 Department of Surgery, Hiroshima University School of Medicine, Hiroshima, Japan; 3 Department of Human Genome Research, Kazusa DNA Research Institute, Kisarazu, Chiba, Japan; 4 Laboratory for Developmental Genetics, RIKEN Research Center for Allergy and Immunology, Yokohama, Japan; 5 Core Research for Evolutional Science and Technology, Japan Science and Technology Agency, Yokohama, Japan; Northern Institute for Cancer Research, United Kingdom

## Abstract

USP21 is a deubiquitylase that catalyzes isopeptide bond hydrolysis between ubiquitin and histone H2A. Since ubiqutylated H2A (ubH2A) represses transcription, USP21 plays a role in transcriptional activation. On the other hand, the localization of USP21 suggests it has an additional function in the cytoplasm. Here, we identified a USP21 short variant (USP21SV) lacking a nuclear export signal (NES). Differential localization of USP21SV, more in the nucleus than the USP21 long variant (USP21LV), suggests they have redundant roles in the cell. Ectopic expression of both USP21 variants decreased ubH2A in the nucleus. Furthermore, both recombinant USP21 variants activate transcription by deubiquitylating ubH2A *in vitro*. These data suggest multiple roles for USP21 in the ubiquitylation-deubiquitylation network in the cell.

## Introduction

Eukaryotic genomic DNA interacts with numerous proteins to form the higher order structure chromatin. Packaging of the genomic DNA into chromatin appears to affect levels of gene transcription. Eukaryotic gene expression is regulated by chromatin structure together with the cellular network of cis-acting elements and trans-acting factors [1]–[3]. The fundamental unit of chromatin is the nucleosome which is composed of 147 base pairs (bp) of DNA wrapped 1.65 turns around the histone octamer of the four core histones (H2A, H2B, H3, and H4) [4]. Nucleosomes act as regulators of multiple stages of transcription, including initiation, elongation, and termination. Diverse post-translational modifications of the nucleosomal histone tail play roles in controlling the structure and function of chromatin [2], [5], [6]. Among these diverse histone modifications, histone ubiquitylation has emerged to play roles in transcriptional regulation [7], [8].

Histone H2A was the first protein found to be ubiquitylated using the hepatocyte regeneration model [9], [10]. The liver retains the capacity to regenerate in response to changes in mass or function in both humans and animals. Following a two thirds hepatectomy, normally quiescent hepatocytes undergo one or two rounds of replication to restore the liver mass. A large number of genes comprise the regulatory network involved in liver regeneration [11]–[14]. The amount of monoubiquitylated core histone H2A changes dramatically around core promoters during hepatocyte regeneration [7]. Since histone H2A deubiquitylase should be activated during hepatocyte regeneration, we found that USP21 catalyzed the hydrolysis of mouse liver chromatin ubH2A based on expression array data of the regenerating liver. USP21 catalyzed the hydrolysis of nucleosomal ubH2A but not free ubH2A in solution [7]. Furthermore, we found that USP21 activates transcript initiation using *in vitro* reconstituted chromatin [7], [15], [16].

On the other hand, receptor-interacting protein 1 (RIP1) was found to be another substrate of USP21. RIP1 plays an important role in the positive and negative regulation of tumor necrosis factor α (TNFα)-induced nuclear factor κB (NF-κB) activation. Thus, it has been considered that USP21 plays a role in the regulation of TNFα-induced NF-κB activation indirectly by deubiquitinating RIP1 [17]. Furthermore, it was found that USP21 is unique in showing clear association with both centrosomes and microtubules. Using an *in vitro* assay, it was shown that microtubule binding is direct and identified a novel microtubule binding motif encompassed within the amino acids 59–75 of the N-terminus of USP21. It was concluded that USP21 plays a role in the governance of microtubule- and centrosome associated physiological processes [18].

Here, we identified a USP21 short variant (USP21SV) lacking NES. Differential localization of USP21SV in the nucleus more than the USP21 long variant (USPLV), suggests they have redundant roles in the cell.

## Results

### Detection and cloning of USP21 SV

**Figure 1:**
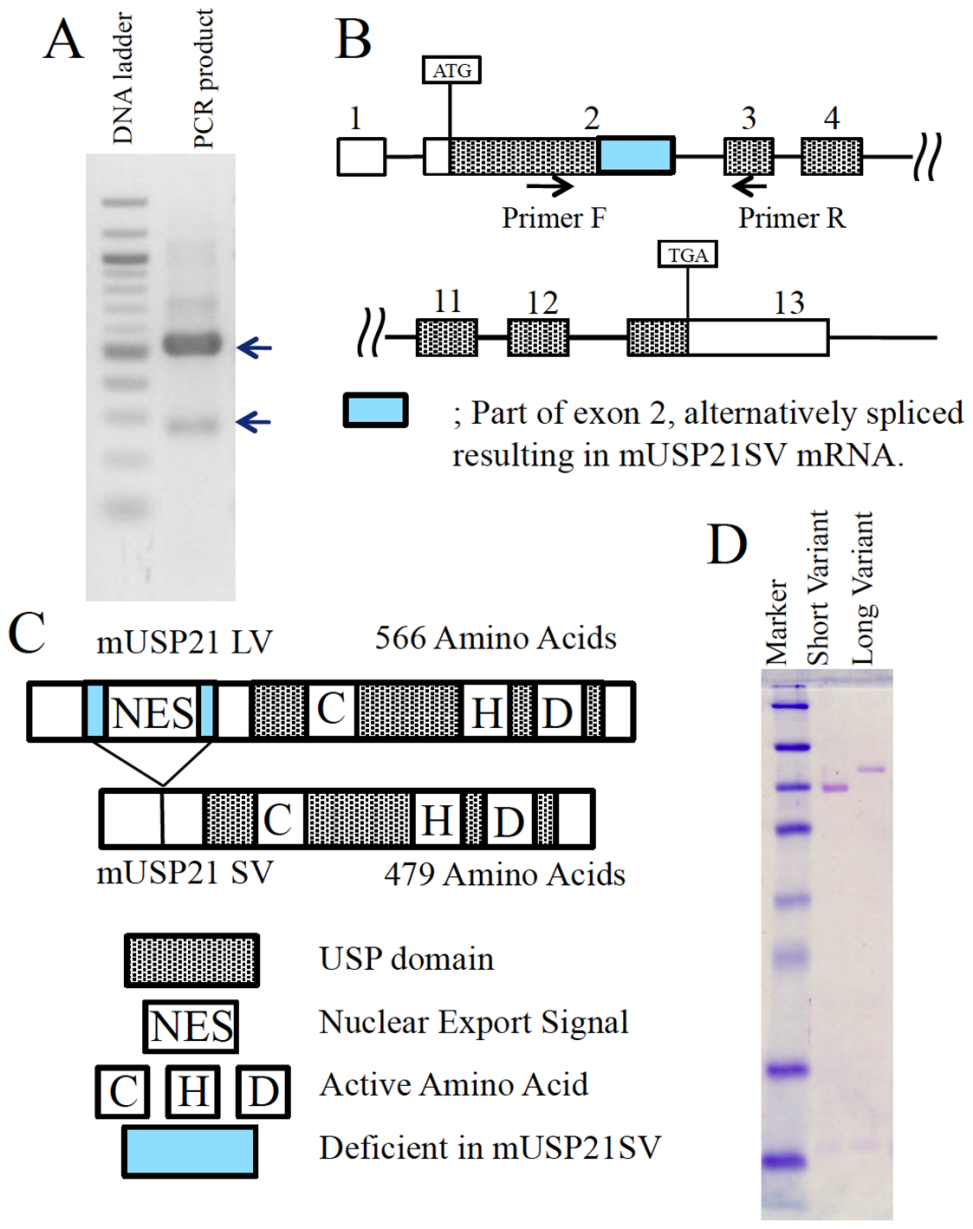
Identification of the USP21SV without Nuclear Export Signal. A) Total RNA from 10-week-old mouse liver was analyzed by RT-PCR using USP21 gene-specific primers. Two different sized PCR products were detected as denoted by the arrow. B) Design of the specific primers located in the USP21 gene. C) Schemes of USP21LV and USP21SV. The alternatively spliced sequence in USP21SV is indicated with the blue box. D) Coomassie staining of Usp21SV and Usp21 LV.

**Figure 2:**
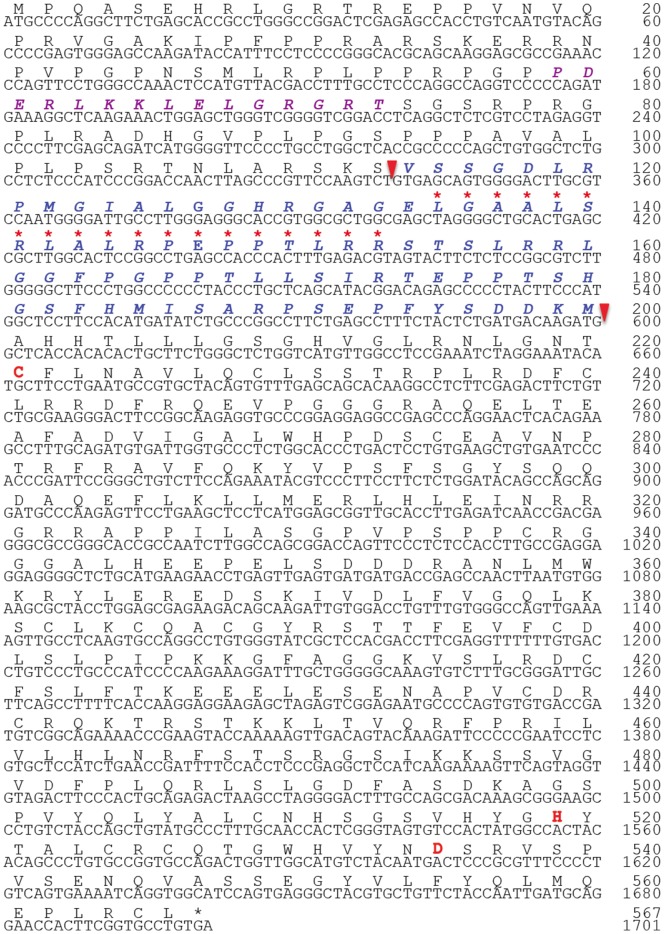
Sequences of the long and short variant of USP21. Nuclear export signal. (NES) is indicated with a red asterisk. USP21SV is deficient in part of exon 2, comprising an 87 amino acid sequence with a bold italic blue letter between two arrowheads. Active site residues are indicated with a bold red letter. Estimated microtubule binding sites are indicated with a bold italic purple letter.

We identified that USP21 deubiquitylates nucleosomal ubH2A in the nucleus and activates transcription [7]. *In vitro*, we found that ubH2A inhibits H3 lysine 4 methylation and proved that USP21 activates transcript initiation by permitting H3 lysine 4 methylation through deubiquitylating ubH2A [7]. On the other hand, it was reported that USP21 is located in the cytoplasm and plays a role in the governance of microtubule- and centrosome associated physiological processes [18]. To clarify these two possible roles both in the nucleus and cytoplasm, we tried to analyze the expression pattern and localization of USP21 in more detail. First, we tried to analyze the expression pattern of USP21 using RT-PCR. We noticed that a small transcript was reproducibly amplified using multiple pairs of primers. PCR product that suggests alternative splicing and PCR primer used for its amplification are shown in Fig. 1A and Fig. 1B respectively. We speculated that there is alternative splicing and subsequently amplified the coding region of USP21LV and USP21SV. We sequenced the coding region of the USP21SV and deposited its sequence to GenBank (accession number is KF646669). Subsequently, we subcloned USP21SV into pET15 expression plasmid with a His-tag resulting in pETHisUSP21SV and confirmed the sequences of the USP21SV. USP21LV have already been reported [7], [18] with nuclear export signal (NES) as illustrated in the Fig. 1C scheme indicated with a red asterisk in Fig. 2 (GenBank accession number of USP21LV is NM_013919). USP21SV is a splicing variant without part of exon 2 indicated with a blue box in Fig. B, C and an 87 amino acid sequence in blue bold italic letters between two arrowheads in Fig. 2. Thus USP21SV does not contain NES affecting cellular localization. Active site residues that are well conserved among serine proteases are indicated with a bold red letter in Fig. 2.

### Recombinant USP21SV deubiquitylated ubH2A and activated transcription in vitro

**Figure 3:**
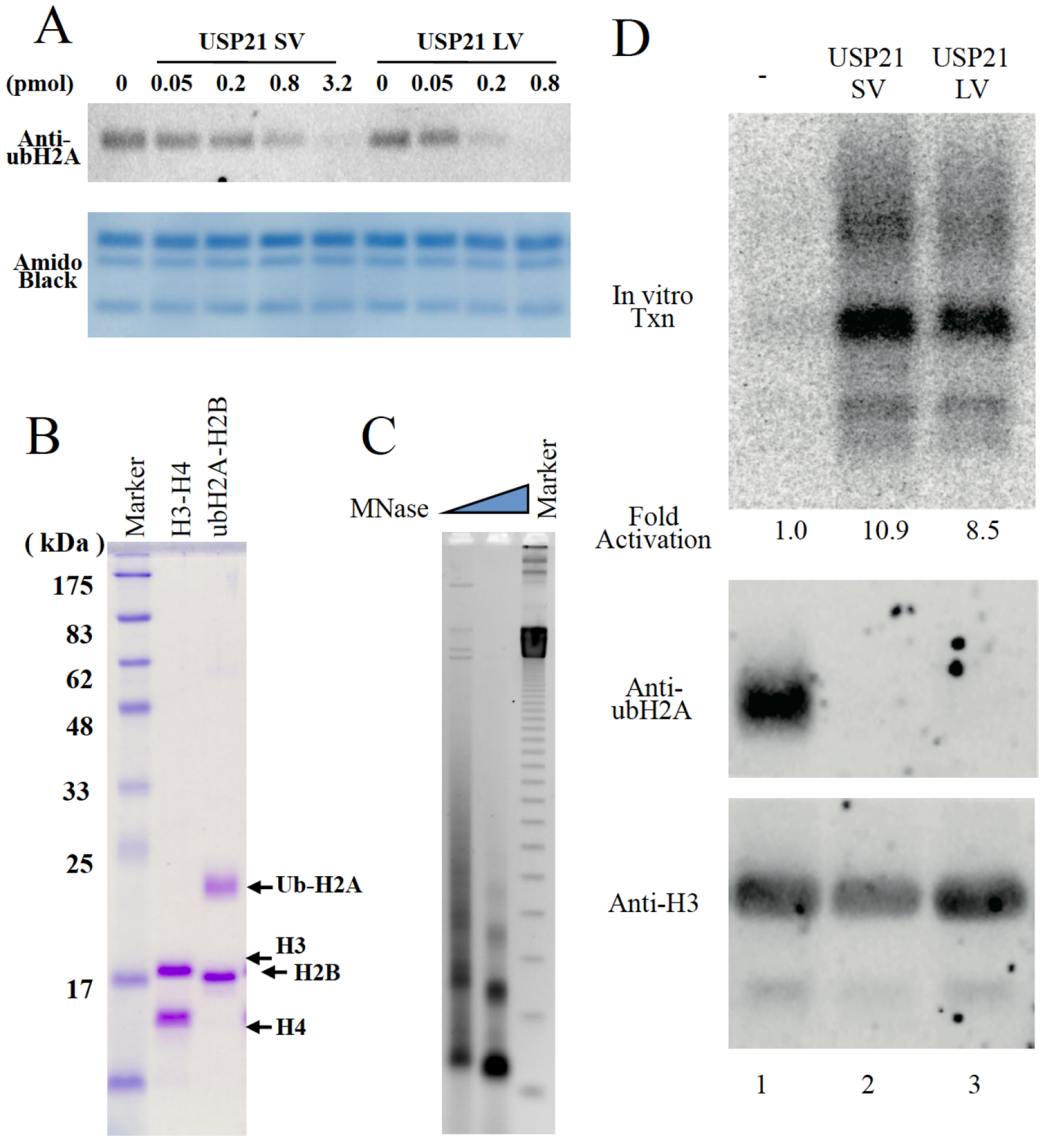
Both USP21SV and USP21LV deubiquitylate ubH2A and activate transcription. A) Deubiquitylation assay. Different amounts of recombinant USP21SV and USP21LV were incubated with native chromatin as indicated. Signals from ubH2A were detected by Western blotting using anti-ubH2A and Alexa 647-Protein A (Life Technologies). Amido black staining was used as loading control. B) Native H3-H4 and ubH2A-H2B were purified from mouse liver. C) Chromatin was assembled by salt dialysis and digested with micrococcal nuclease. D) Ubiquitylated chromatin was subjected to transcription after treatment with USP21-SV, USP21-LV or control buffer as indicated. Deubiquitylated H2A was confirmed by Western blotting with anti ubH2A. Transcripts were detected by primer extension.

We expressed both USP21SV and USP21LV using E. Coli and purified the recombinant protein with a His tag. Both of them were expressed and purified to the same extent (Fig. 1D). We tested the activity of the purified recombinant USP21SV and USP21LV. Both variants can hydrolyze the isopeptide bond between histone H2A and ubiquitin (Fig. 3A). The observation that the ubH2A signal is not detected anymore in the presence of 0.8pmol of USP21LV, whereas a shadow is still observed in the presence of 3.2pmol of USP21SV suggested that USP21SV is less active compared to USP21LV using native chromatin as the substrate. Then we tested the activity using an *in vitro* transcription assay. First, we assembled chromatin using the native purified H3–H4 tetramer and ubiquitylated H2A–H2B dimer (Fig. 3B). Chromatin reconstitution is mediated by salt dialysis and revealed by micrococcal nuclease. Ubiquitylated H2A–H2B can assemble regularly spaced nucleosomes together with H3–H4 tetramers (Fig. 3C). Using this chromatin we analyzed both USP21SV and USP21LV to see if they affect transcription *in vitro*. Consistent with our previous report [7], ubiquitylated H2A inhibits transcription detected by primer extension as shown in Fig. 3D lane 1. Both USP21SV and USP21LV can deubiquitylate ubH2A in the nucleosome as shown by Western blotting and activate transcription as indicated by primer extension in Fig. 3D lanes 2, 3.

### USP21SV localizes more in the nucleus than USP21LV

**Figure 4:**
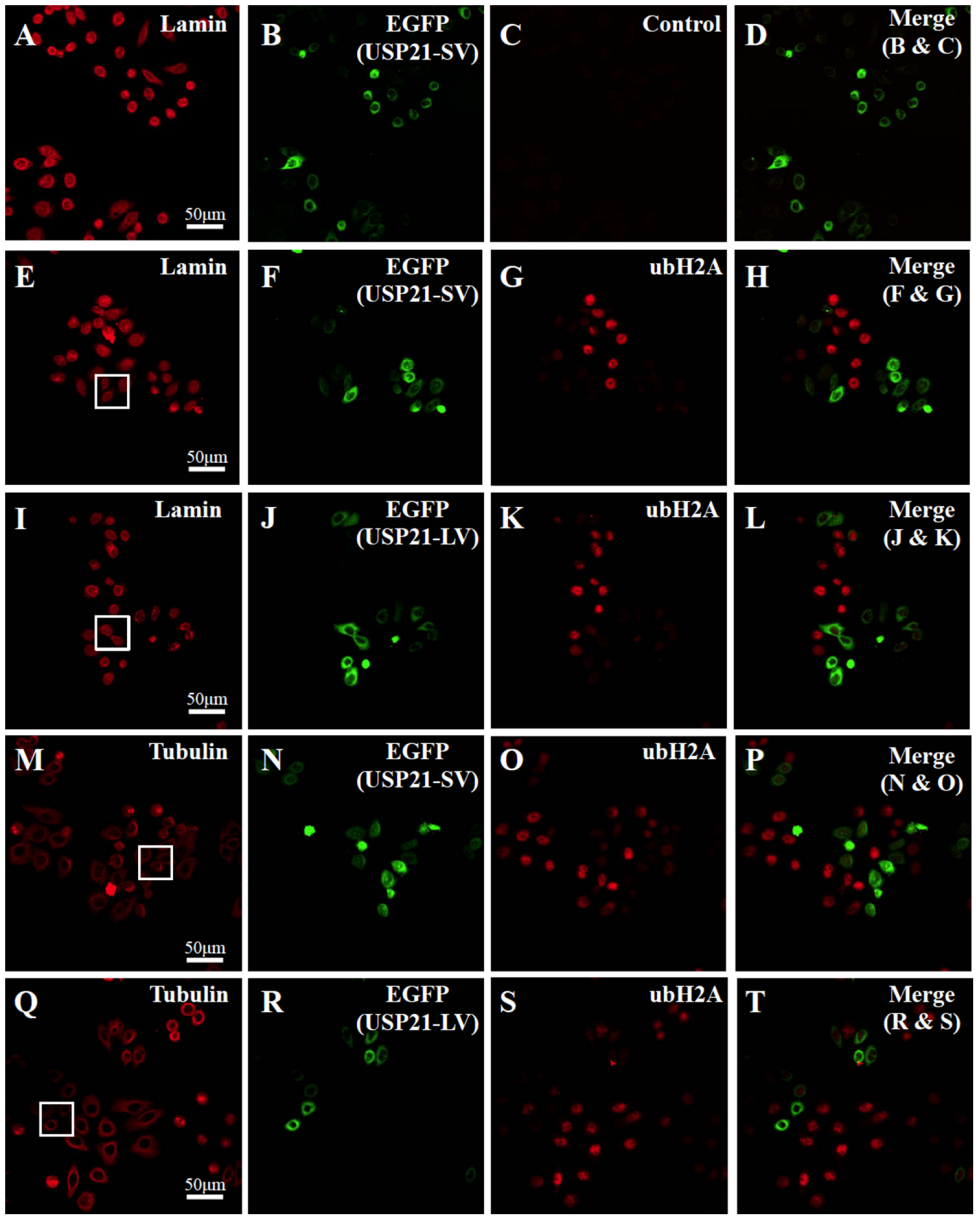
ubH2A signal decreased when both USP21 variants are over-expressed. Hela cells were transfected either with EGFP-USP21SV (A–H, M–P) or EGFP-USP21LV (I–L, Q–T) for 24 hours. Anti-lamin antibody was used for immunofluorescence identification of the nucleus (A, E, I) and anti-tubulin antibody was used for immunofluorescence identification of the cytoplasm (M, Q). Anti ubH2A antibody was used to evaluate nuclear ubH2A (G, K, O, S). Control IgG was used as a negative control for immunofluorescence of ubH2A (C). The merged image illustrates the relationship of either EGFP-USP21SV (H, P) or EGFP-USP21LV (L, T) expression and ubH2A. Merging of Control and GFP showed features of the cell with GFP but without H2A ubiquitylation (D). Scale bar indicated 50 μm.

**Figure 5:**
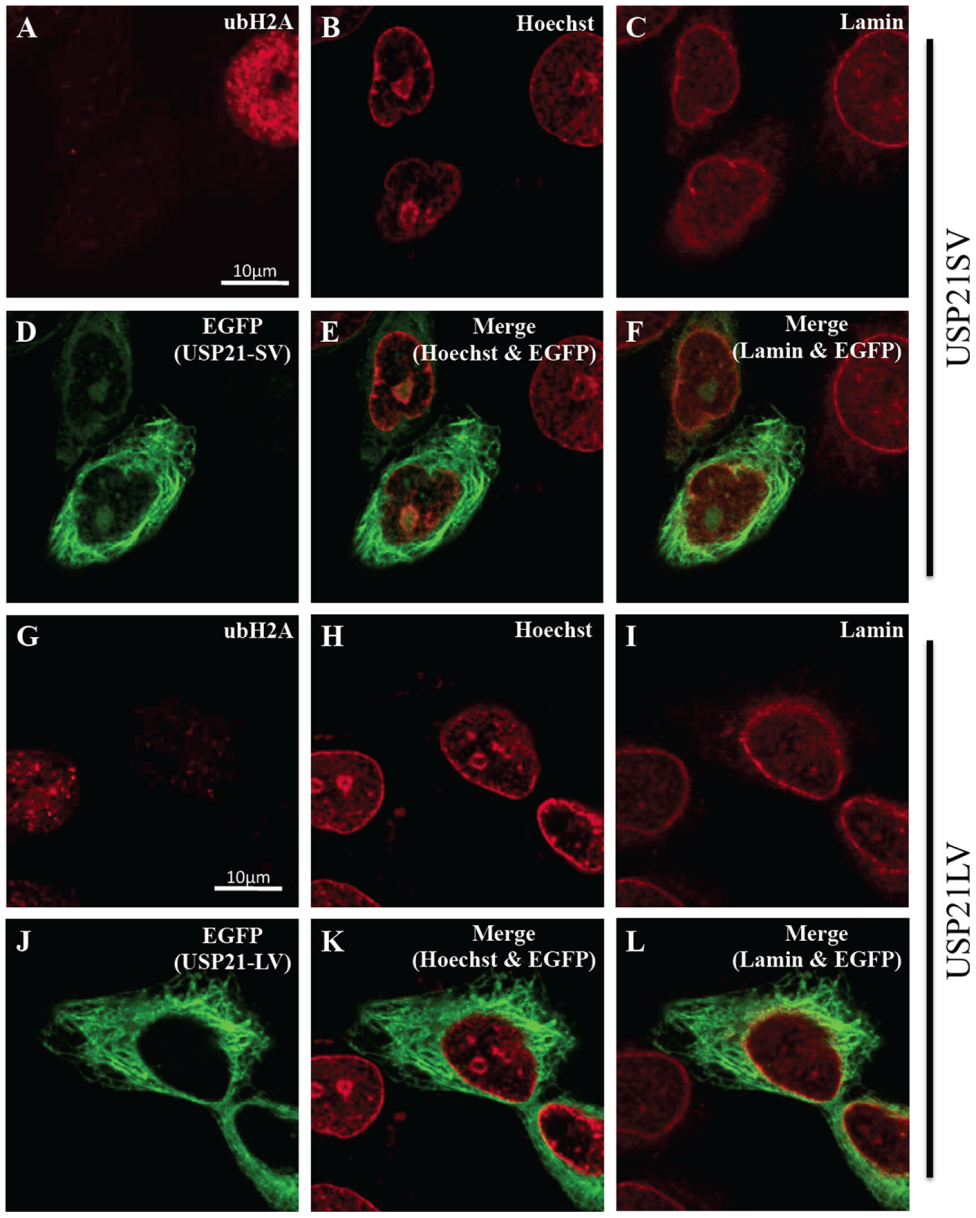
USP21SV localizes more in the nucleus than USP21LV. Detailed analysis of the box in Fig.–F and G–L, respectively. Merger of B & D, C & D, H & J, I & J is shown in E, F, K, L, respectively. Scale bar indicated 10 μm.

**Figure 6:**
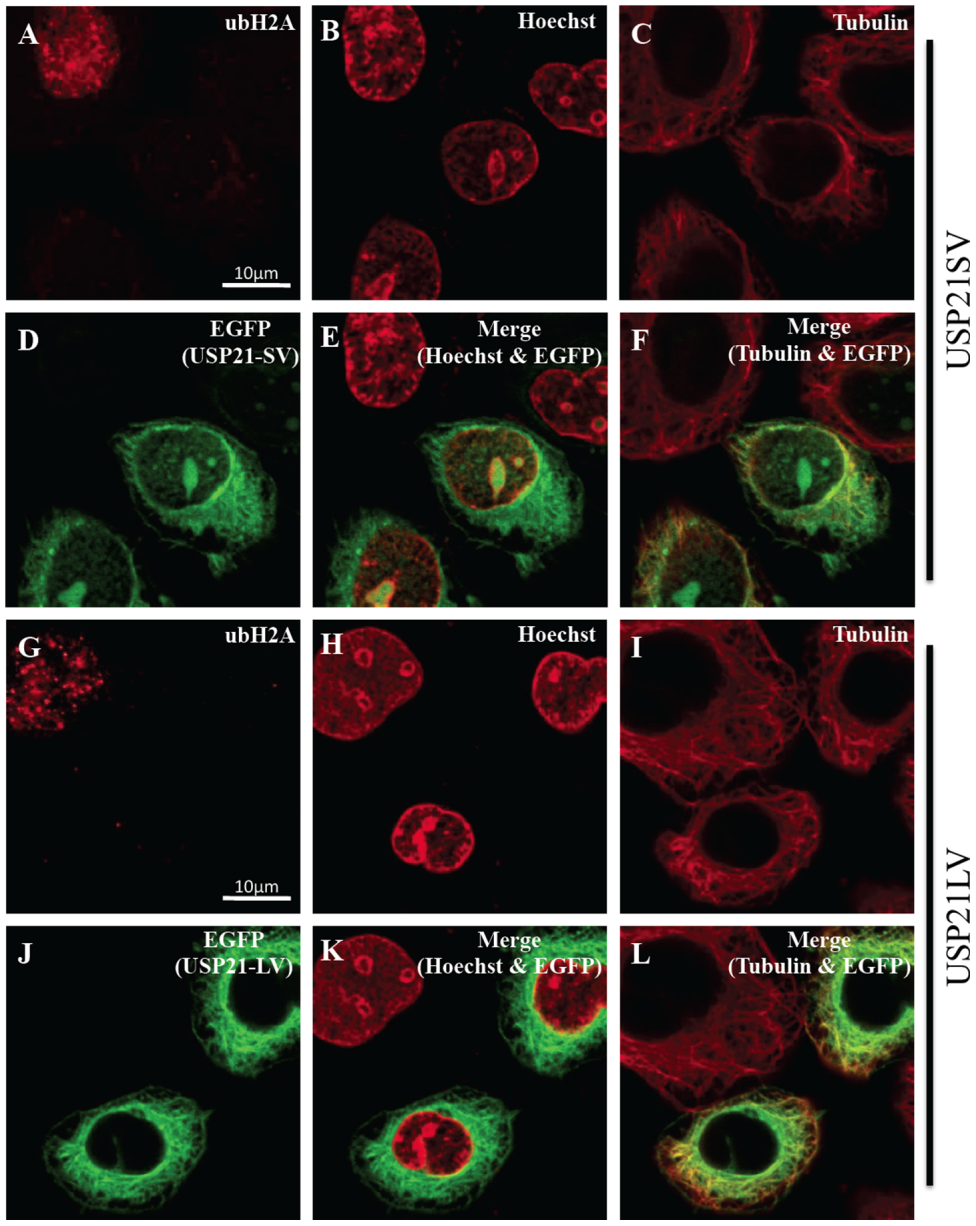
USP21LV localizes mainly in the cytoplasm. Detailed analysis of box in Fig.–F and G–L respectively. Merger of B & D, C & D, H & J, I & J is shown in E, F, K, L, respectively. Scale bar indicated 10 μm.

Redundant roles of USP21 are speculated from previous observation and reports [7], [18]. Since we identified USP21SV without NES, we were interested in the differences between USP21SV and USP21LV. An immuno-fluorescence experiment was performed to clarify the detailed location of both USP21SV and USP21LV using the Hela cell line. Control IgG for the autofluorescence of ubH2A showed negative fluorescence and merging of Control and GFP showed features of the cell with GFP but without H2A ubiquitylation (Fig. 4A–D). Ectopic expression of USP21SV or USP21LV and histone H2A ubiquitylation was examined (Fig. 4E–T). Judging from the merger of ubH2A and EGFP (Fig. 4H, L, P, T), it is clear that ubH2A signal decreased when both USP21 variants are over-expressed. Subsequently, we examined the fine localization of both variants with enlarged images of the box in Fig. 4E, I, M, Q which correspond to Fig. 5A–F, Fig. 5G–L, Fig. 6A–F, Fig. 6G–L, respectively. USP21SV is localized to the nucleus, nuclear membrane and cytoplasm (Fig. 5A–F, Fig. 6A–F). USP21LV is mainly located in the cytoplasm and excluded from the nucleus. It is also located in the nuclear membrane which was revealed by merging of tubulin, Hoechst or lamin and USP21LV (Fig. 5G–L, Fig. 6 G–L,).

### Ectopic expression of both USP21 variants decrease nuclear ubH2A

**Figure 7:**
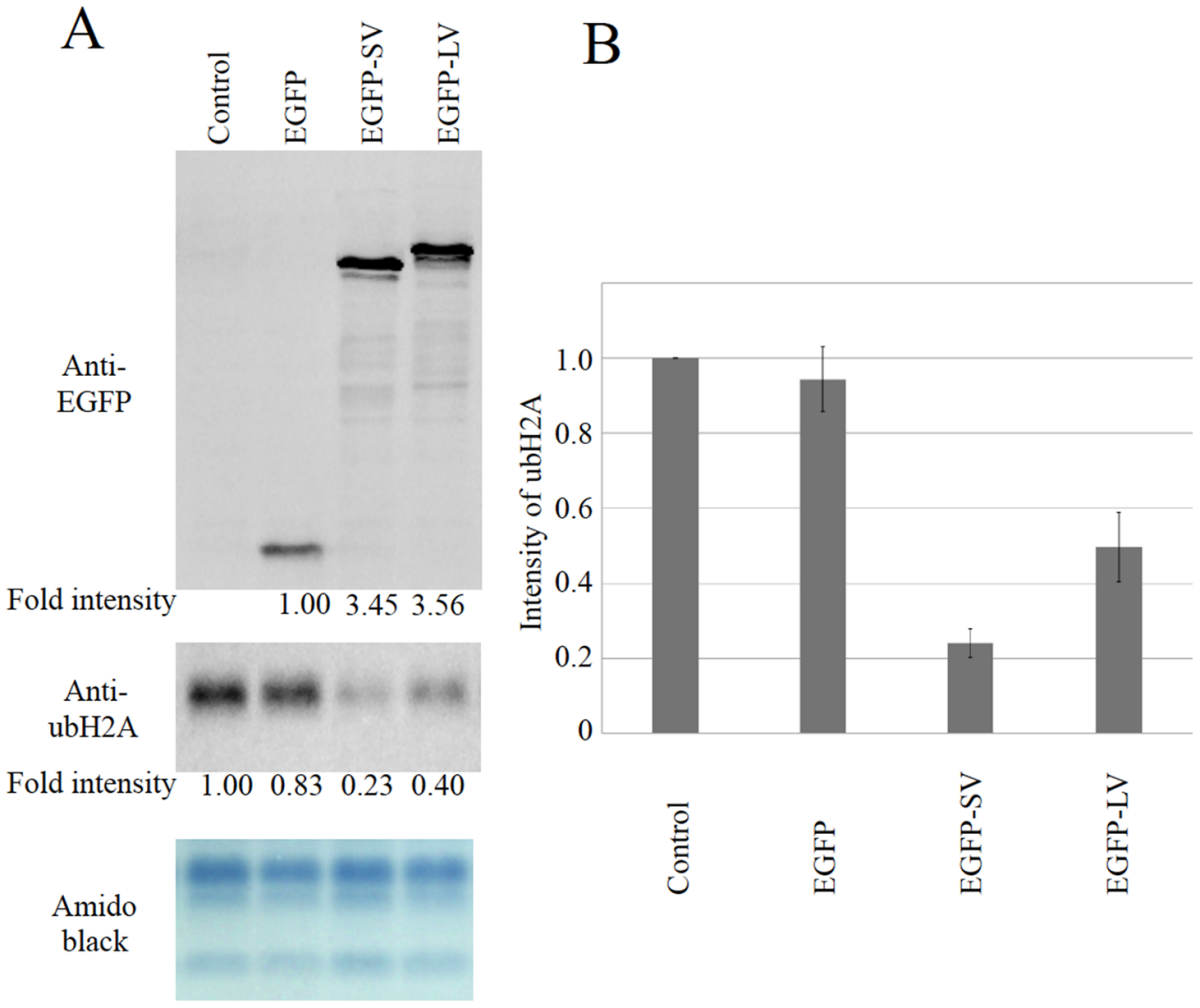
USP21SV is more active than USP 21LV in vivo. 293T cells were transfected either with EGFP-USP21SV, EGFP-USP21LV or EGFP as indicated. Lipofectamine LTX & PLUS treatment was used as a control. After 24 hours cells were subjected Western blot analysis. A) Anti-EGFP was used for assessment of USP21 introduction and amido black was used for loading control. Quantification of ubH2A was used to evaluate activity of USP21 variants *in vitro*. B) Intensity of ubH2A was measured using three independent transfection experiments. The intensity of ubH2A is in arbitrary units compared to the value of the control.

To confirm the decrease of ubH2A in the nucleus, we introduced both USP21SV and USP21LV into 293T cells. We measured ubH2A using Western blot analysis. The intensity of ubH2A in three independent experiments indicated that ubiquitylated H2A decreased more when USP21SV was introduced compared to USP21LV (Fig. 7A, B). Although USP21LV is more active than USP21SV *in vitro*, USP21SV seems to be more active compared to USP21LV *in vivo*.

## Discussion

Gene expression requires the temporal and spatial assembly of the transcriptional regulatory machinery. Transcription is a dynamic genomic process that requires an underlying structural organization in the cell nucleus [19], [20]. *In vitro*, we proved chromatin dynamics showing that histone acetylation facilitates the loss of H2A–H2B dimers from chromatin resulting in alteration of the nucleosome structure [21]. *In vivo*, it has been proven that the inner core of the nucleosome is very stable, whereas H2A–H2B on the surface of active nucleosomes exchanges continually [22]. These previous observations well explain that USP21 localized to the nuclear membrane can deubiquitylate the whole core ubiquitylated histone H2A in the nucleus even if USP21 is not colocalized with ubH2A because histone H2A–H2B dimers exchange continuously due to chromatin dynamics.

The subcellular localization of USP21 also provides clues to cellular processes. Recently, it was reported that USP21 is localized to microtubules and centrioles using GFP tagged USP21. In addition, USP21 knockdown in PC12 cells inhibits nerve growth factor–induced neurite outgrowth suggesting its role associated with microtubules [18]. To the contrary, we found USP21SV lacking NES was localized to the nucleus more than USP21LV. Both variants being mostly located in the nuclear membrane suggest their role in the nucleus.

The ubiquitylase activity of USP21SV is stronger than USP21LV *in vivo* and USP21LV is more active than USP21SV *in vitro*. This discrepancy is probably due to subcellular localization that suggests functional differences for both variants. In addition, both of them clearly activate transcriptional initiation as shown in Fig. 3D. Since USP21SV is located more in the nucleus, it plays a role related to events in the nucleus. Thus, we propose a dual function of USP21, one is a role for cytoskeleton organization related to microtubules and the other is a role for transcriptional activation.

Both variants play a role with some redundancy judging from overlapping subcellular localization and function. Ubiquitylation is an essential posttranslational modification involved in diverse cellular processes, including membrane trafficking, cell division, cell signaling, and transcription. It is suggested that USP21 may play multiple roles in diverse cellular processes by deubiquitylating diverse substrates.

## Materials and Methods

### Ethics Statement

All animal experiments in this study strictly followed a protocol approved by the Institutional Animal Care and Use Committee of Nagasaki University (approval number: 1008060874-2).

### RNA extraction and RT-PCR

Total RNA was extracted from 10-week-old mouse liver using ISOGENII (Nippon Gene). cDNA was synthesized from 1 μg of total RNA using random hexamers (Takara) and oligo dT primers (Life Technologies), and reverse transcriptase (NEB). The cDNA was used as a template for RT-PCR using gene-specific primers. Fragments were PCR amplified by a 2 min melting step at 94°C, followed by 35 cycles of amplification (94°C for 30 sec, 55°C for 45 sec, and 72°C for 90 sec) and a terminal 5 min extension at 72°C.

Forward primer: GATGAAAGGCTCAAGAAACTGGAG.

Reverse primer: TGCTGCTCAAACACTGTAGC.

### Immunofluorescence study

Hela cells were transfected with 250 ng of pEGFP-USP21LV or pEGFP-USP21SV using Lipofectamine LTX & PLUS Reagent (Life Technologies) according to the user's manual (Lipofectamine LTX & PLUS Reagent protocol 2013). One day after transfection, cells were fixed in −30°C methanol for 4 minutes. Hela cells were incubated with anti-Lamin B2 mouse monoclonal antibodies (1∶200 dilution, Santa Cruz sc-56147), anti-α-Tubulin mouse monoclonal antibody (1∶1000, Sigma T5168) and purified rabbit polyclonal anti-ubH2A antibodies [7]. After washing, signals were detected by Alexa Fluor 568-conjugated goat anti rabbit IgG antibodies (1∶800, Life Technologies A11011) and Alexa Fluor 633-conjugated goat anti mouse IgG antibodies (1∶250, Life Technologies A21050). DNA was stained with Hoechst 33342 (1∶2000). Images were collected using a deconvolutional microscope (OLYMPUS FV1000D IX81).

### Purification of recombinant USP21

DNA fragments encoding USP21SV and USP21LV were subcloned into the pET15His-tag expression vector generating pETHisUSP21SV and pETHisUSP21LV, respectively. USP21SV and USP21LV were expressed in BL21DE3 E. coli and purified with the His-tag. Both of tag purified USP21 variants were further loaded onto SP sepharose. After washing the SP sepharose with 0.1M KCl-HEG buffer, the proteins bound to SP sepharose were eluted by a 0.2M–1.0M KCl-HEG gradient. Purified preoteins were dialyzed against 50 mM KCl-HEG buffer.

### Deubiquitylation assay

Mouse liver chromatin 2 μg was incubated with recombinant USP21 (0.05–3.2 pmol) and 1 mM MgCl_2_ for 15 min at 37°C. After incubation, the reaction was stopped by SDS sample buffer and subjected to SDS-polyacrylamide gel electrophoresis (SDS-PAGE), and transferred onto nitrocellulose membranes (Bio-Rad). Primary antibody used for Western blotting was anti-ubH2A antibody [7] and anti-H3 antibody. For detection of the signal, Alexa 647-Protein A (Life Technologies) was used. The membrane was scanned by a Typhoon Variable Scanner (GE Healthcare) using an excitation laser at 635 nm with an emission filter at 665 nm.

### Purification of core histones

For the purification of the ubH2A-H2B dimer, mouse liver was homogenized and digested with micrococcal nuclease. This preparation was fractionated on a 15%–40% (w/v) glycerol gradient, and the peak fraction was applied to a hydroxyapatite column. Core histones were eluted with a linear salt concentration gradient and divided into H3–H4 dimers. The ubH2A-H2B dimer was purified using the anti-ubH2A antibodies. These antibodies could immunodeplete the ubH2A-H2B dimer from the bulk H2A–H2B dimer. The ubH2A-H2B dimer was eluted from an affinity column using a bifurcated peptide. The purified H2A–H2B and ubH2A-H2B dimers were further purified by SP sepharose column chromatography.

### Reconstitution of nucleosomes by salt analysis

Typically, chromatin was reconstituted with 100 μg of supercoiled pGIE0 plasmid DNA, which has GAL4-binding sites before the AdE4 promoter, and 100 μg of purified *Drosophila* core histones, using salt dialysis techniques [23], [24]. The fully reconstituted chromatin was then enriched by 15%–40% (w/v) glycerol gradient sedimentation (60,000 rpm; 6 hours; 4°C; Beckman SW60 rotor). The gradient fractions containing the fastest migrating chromatin (which had the highest density of nucleosomes) were pooled and dialyzed against HEG (25 mM Hepes (pH 7.6), 0.1 mM EDTA and 10% glycerol) buffer containing 50 mM KCl.

### In vitro transcription

For *in vitro* transcription, 50 ng of pGIE0 plasmid DNA was assembled into chromatin by salt dialysis was subjected to *in vitro* transcription by adding nuclear extract and 500 nM of each NTP. Transcripts were detected by primer extension and subsequent 8% denaturing gel electrophoresis. A primer that is located 83 nucleotides from the initiation site was used for a primer extension reaction.

### Generation of anti-EGFP antibodies

DNA encoding full length EGFP was subcloned into the pGEX6P-2 expression vector, expressed in BL21DE3 E. coli, and purified with the GST-tag. Five rabbits were immunized with 1.5 μg purified EGFP protein together with Freunds Complete adjuvant for the first two immunizations and Freunds Incomplete adjuvant after the third. After the fourth immunization, serum was collected and incubated with antigen coupled Affigel 10 (Bio-rad). EGFP specific antibody binding with Affigel 10 was eluted by 0.2M Glycine buffer (pH2.2) and neutralized by 1.5M Tris-HCl (pH8.5). Eluted antibody was precipitated in 65% ammonium sulfate and the resolved pellet was dialyzed against HEG buffer containing 50 mM KCl. Specificity of purified rabbit polyclonal anti-EGFP antibody was confirmed by Western blotting. Only EGFP over expressed sample could detect the EGFP signals (Fig. 7A).

### Ectopic expression of USP21 in 293T cells

293T cells were transfected with 2.5 μg EGFP-USP21LV or pEGFP-USP21SV in 6 well dish, using Lipofectamine LTX & PLUS Reagent (Life Technologies) according to the user's manual (Lipofectamine LTX & PLUS Reagent protocol 2013). At 1 day after transfection, cells were collected with SDS sample buffer and subjected to SDS-polyacrylamide gel electrophoresis (SDS-PAGE), and transferred onto nitrocellulose membranes (Bio-Rad). Membranes were stained with 1% amido black solution to detect the core histones. Primary antibody used for Western blotting was anti-ubH2A antibody [7] and purified rabbit polyclonal anti-EGFP antibodies. For detection of the signal, Alexa 647-Protein A (Life Technologies) was used. The membrane was scanned by a Typhoon Variable Scanner (GE Healthcare) using an excitation laser at 635 nm with an emission filter at 665 nm.
